# Supplementary material for: Aging Triggers H3K27 Trimethylation Hoarding in the Chromatin of Nothobranchius furzeri Skeletal Muscle
Source: Cells. 2019 Sep 28;8(10):1169. doi: 10.3390/cells8101169 (PMC6829443; doi:10.3390/cells8101169)
Supplement: Supplementary file 1 [file cells-08-01169-s001.pdf]

Supplementary figures and table

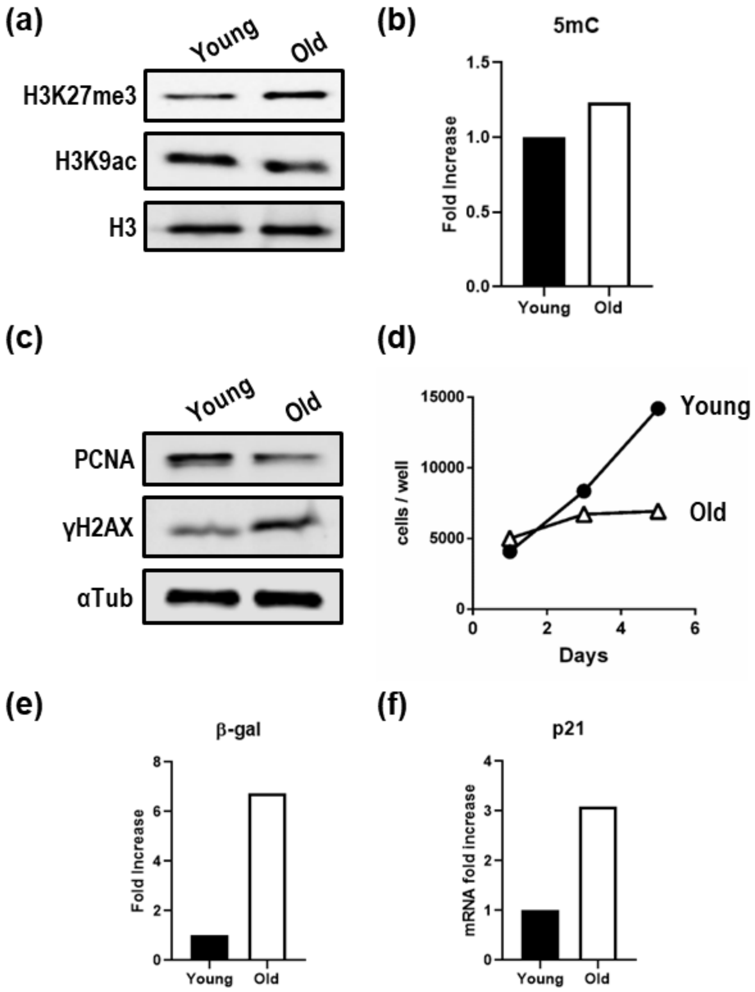

Figure S1. Cells derived from *Nfu* skeletal muscle tissues resemble tissue of origin. (**a**) Western blot for H3K27me3 and H3K9ac in cells isolated from young and old *Nfu* skeletal muscle tissue. Tissues from multiple animals were pooled for cell isolation. Total histone 3 was used as loading control. (**b**) Global DNA methylation quantification of 5mC in cells isolated from young (black bar) and old (white bar) muscle tissue expressed as fold-change versus young. (**c**) Western blot for proliferating antigen (PCNA) and γ histone 2AX (γH2AX). α-Tubulin (αTub) was used as loading control. (**d**) Growth curve related to cells derived from young (black circles) and old (white triangles) *Nfu* skeletal muscle tissue. (**e**) Analysis of β-galactosidase (β-Gal) staining in young and old *Nfu* skeletal muscle tissue, number of positive cells expressed as fold increase versus young sample. (**f**) qRT-PCR analysis of p21 in cells derived from young and old cells derived from *Nfu* muscle tissue.

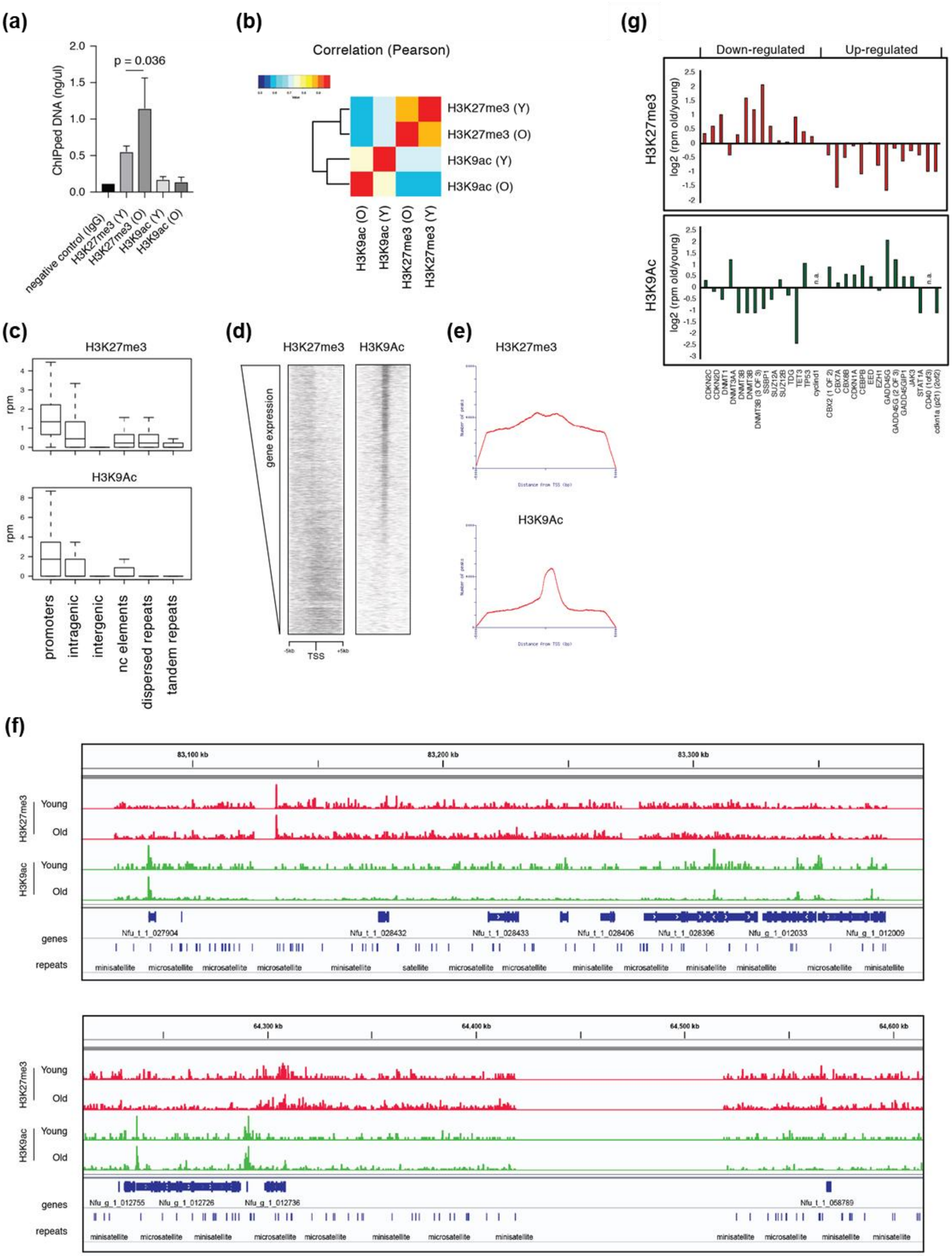

Figure S2. Histone marks H3K27me3 and H3K9ac cluster at the transcription start sites. (**a**) Amount of immune-precipitated DNA (ng/μl) using anti-H3K27me3 and anti-H3K9ac antibodies. P-value is calculated using t-test. (**b**) Person correlation of mapped reads revealed separation of H3K27me3 and H3K9ac samples. (**c**) Distribution of

H3K27me3 (upper plot) and H3K9ac (bottom plot) signals in different genomic features. RPM = Read Per Million. (**d**) Heatmap of H3K27me3 and H3K9ac signal distribution around transcription start sites (TSS). The genes are sorted from high to low expression. (**e**) Distribution of H3K27me3 and H3K9ac peaks (p-value cutoff = 0.001) around the TSS. (**f**) Large genomic views of the mapped reads in the indicated conditions. (**g**) Log2 of the old/young ratio of H3K27me3 (upper panel) and H3K9ac (bottom panel) signal intensity (RPM) for some differentially expressed genes in RNA level during aging.

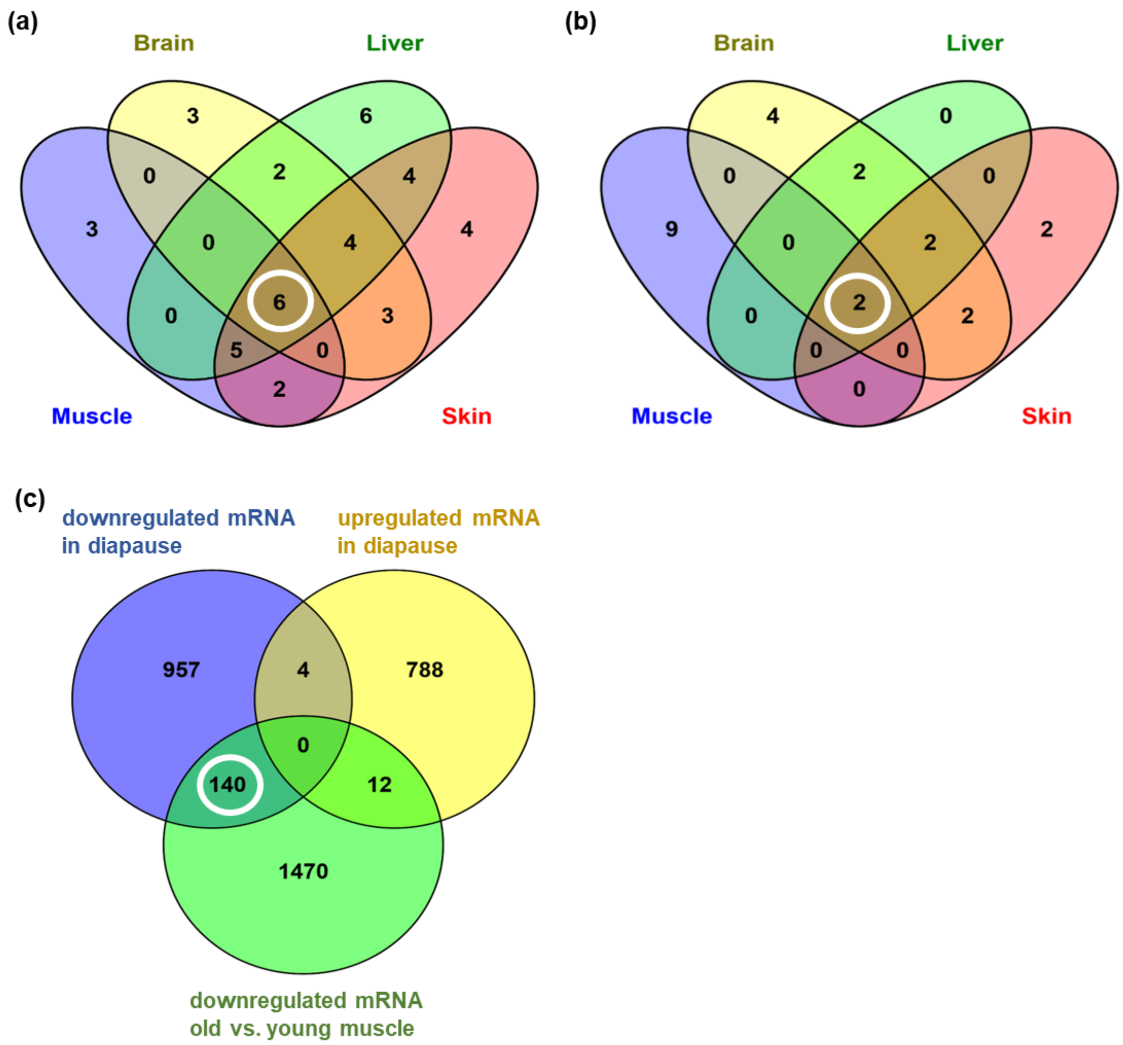

Figure S3. Common mRNA expression profiles in different types of *Nfu* tissues during aging and during diapause indicate global trends. (**a**) Venn diagram depicting KEGG pathways commonly down-regulated in *Nfu* skeletal muscle (blue), brain (yellow), liver (green) and skin (red) tissue comparing young versus old samples. Six KEGG pathways shared by all four tissues were found (white circle): Ribosome biogenesis in eukaryotes, DNA replication, cell cycle, aminoacyl-tRNA biosynthesis, pyrimidine metabolism and RNA transport. (**b**) Venn diagram depicting up-regulated KEGG pathways in *Nfu* muscle (blue), brain (yellow), liver (green) and skin (red) tissue comparing young versus old samples. Two common KEGG pathways are found (white circle): Jak/Stat signaling and cytokine/cytokine receptor interaction. (**c**) Common mRNA expression profile of down-regulated mRNAs during diapause (blue circle), up-regulated mRNA during diapause (yellow circle) and down-regulated mRNA in old *Nfu* skeletal muscle tissue (green circle): mRNAs of 140 genes are down-regulated during diapause and during aging (white circle). Data from Baumgart *et al*. (2014) (**a** and **b**) and Reichwald *et al*. (2015) (**c**) was used to compare with sequencing results from skeletal muscle.

Table S1. Top 50 regulated transcripts (±1 log2 FC; base-mean ≥5, FDR ≤0.05) in each group depicted in Fig. 4A.

Ensembl gene id	Ensembl chromosome	Ensembl start	Ensembl stop	Ensembl strand	Ensembl gene
**824641**	GapFilledScaffold_327	63.128	68.488	-	KLF9
**649160**	GapFilledScaffold_2177	22269	31055	+	UCP2
**17780**	GapFilledScaffold_369	123399	137173	-	CSRNP1B
**605031**	GapFilledScaffold_1971	82042	100822	+	ZGC:56306
**826927**	GapFilledScaffold_626	50793	73608	-	ZBTB16B(1of2)
**581036**	GapFilledScaffold_4409	19853	46452	+	RASGEF1B(1of2)
**673243**	GapFilledScaffold_123	254595	263808	+	SIK1
**871495**	GapFilledScaffold_5595	16075	32476	-	SOGA3B
**128543**	GapFilledScaffold_288	26344	27228	+	CEBPD
**966928**	GapFilledScaffold_8767	21001	24664	+	MYH1(2of3)
**990605**	GapFilledScaffold_3440	67537	68302	+	NFURLNR03440010010
**939321**	GapFilledScaffold_1269	106127	112760	-	ARRDC2
**531846**	GapFilledScaffold_146	160532	199879	+	CNKSR1
**867431**	GapFilledScaffold_610	39908	57370	+	PDE1B
**957706**	GapFilledScaffold_669	230595	237360	+	ARRDC3A
**674222**	GapFilledScaffold_1753	24533	29586	+	ZGC:65851
**843934**	GapFilledScaffold_3880	34710	37862	-	DDIT4L(2of2)
**859490**	GapFilledScaffold_1547	33392	39398	-	IP6K2(1of2)
**704710**	GapFilledScaffold_1508	67435	76224	-	TIMM44(1of2)
**355284**	GapFilledScaffold_296	126991	162445	+	FNIP1
**519425**	GapFilledScaffold_2446	29759	37279	-	ATAD3B
**962503**	GapFilledScaffold_73	138398	144432	+	CSRNP1A
**685688**	GapFilledScaffold_6214	2811	8503	-	SNAP25(3of3)
**484773**	GapFilledScaffold_786	76990	115787	+	TSC22D3(1of3)
**258357**	GapFilledScaffold_983	56714	58723	+	EVA1C
**158041**	GapFilledScaffold_1636	81645	93317	-	PPRC1(2of2)
**962068**	GapFilledScaffold_292	60344	60880	+	IER2(2of2)
**483825**	GapFilledScaffold_769	97927	162287	+	SESN1
**713692**	GapFilledScaffold_2026	60503	83700	+	WHSC1
**476666**	GapFilledScaffold_132	104148	130966	+	NUAK1(1of2)
**784281**	GapFilledScaffold_4434	10385	33583	+	IGF2BP3
**622626**	GapFilledScaffold_5836	1985	50332	+	ANLN(2of2)
**11155**	GapFilledScaffold_2278	2194	10052	-	PPM1G
**614992**	GapFilledScaffold_9391	19963	21362	-	COX4I1(1of2)
**977955**	GapFilledScaffold_25	328261	331963	-	BHLHE40(2of2)
**407941**	GapFilledScaffold_1115	30876	40232	-	SMARCA5
**432733**	GapFilledScaffold_1121	171487	209599	-	RSBN1L(1of2)
**891380**	GapFilledScaffold_483	254685	351282	+	PACS1(1of3)
**507527**	GapFilledScaffold_1568	126217	134624	-	TNNT2D

**17042**	GapFilledScaffold_1355	314328	315203	+	CEBPB
**410982**	GapFilledScaffold_4905	16083	48451	-	MAP1B(2of3)
**261326**	GapFilledScaffold_7258	367	33544	-	SLC1A4
**374730**	GapFilledScaffold_1608	183172	186260	-	KBTBD12
**540930**	GapFilledScaffold_710	96875	100744	+	SERPINH1
**151295**	GapFilledScaffold_66	230082	279694	+	MICAL2A
**620868**	GapFilledScaffold_8948	571	12099	+	SLC16A12(2of2)
**623059**	GapFilledScaffold_216	157217	160467	+	DDX10
**88156**	GapFilledScaffold_700	128485	196324	-	CLTCB
**759680**	GapFilledScaffold_2366	23840	41512	+	CLUHA
**202270**	GapFilledScaffold_11308	10098	13438	-	MYH6(3of3)
**221083**	GapFilledScaffold_175	121.761	145.743	-	COL2A1A
**14897**	GapFilledScaffold_662	19878	42372	-	ERCC5
**199420**	GapFilledScaffold_50	45001	49384	-	NEFMA(1of2)
**603816**	GapFilledScaffold_239	69596	116405	-	TACC2
**78610**	GapFilledScaffold_7876	23922	27838	-	HIST2H2BE(1of9)
**755476**	GapFilledScaffold_607	139260	157105	+	TUT1
**619878**	GapFilledScaffold_5804	23048	26019	-	NFIL3(2of3)
**591963**	GapFilledScaffold_8988	973	22168	+	ASPM(3of3)
**540692**	GapFilledScaffold_3270	69697	74398	+	TBX22
**529223**	GapFilledScaffold_10342	241	11526	+	SMCR8B
**57170**	GapFilledScaffold_603	208114	213028	-	ARX
**728569**	GapFilledScaffold_1832	119461	169869	+	PDLIM5(2of2)
**85341**	GapFilledScaffold_1770	152338	201300	-	PRX(14of16)
**787172**	GapFilledScaffold_508	199111	201571	+	NFURG00508170300
**896111**	GapFilledScaffold_275	51790	63723	+	SPOCK3(1of2)
**837949**	GapFilledScaffold_3238	46505	52882	+	AKT1S1
**170137**	GapFilledScaffold_5518	52596	75496	-	UACA
**20380**	GapFilledScaffold_286	101208	104664	+	NEFH(2of5)
**204713**	GapFilledScaffold_5114	14242	30001	-	PRDM16(1of2)
**605240**	GapFilledScaffold_3572	63024	101479	-	ZNF704(2of3)
**785268**	GapFilledScaffold_14622	1641	5232	+	HEL_DR4(1of4)
**383358**	GapFilledScaffold_3564	88732	90930	-	STMN1(2of3)
**554129**	GapFilledScaffold_549	43104	54733	-	STMN2B
**907981**	GapFilledScaffold_283	514	12636	-	PGAP3
**940728**	GapFilledScaffold_57	99238	138038	+	PDE4DIP(1of3)
**148972**	GapFilledScaffold_254	372519	375577	+	MARCKSB
**541938**	GapFilledScaffold_4754	2640	57452	+	CDKL5(1of3)
**RF00478**	GapFilledScaffold_3672	25535	25803	-	SCARNA6
**511648**	GapFilledScaffold_3084	2451	20062	+	DSCAML1(4of4)
**829769**	GapFilledScaffold_3908	38203	42815	-	ASB15(2of2)
**992166**	GapFilledScaffold_4413	60313	61325	-	NFURLNR04413020010
**537826**	GapFilledScaffold_1951	79724	112717	+	KIF11

**821510**	GapFilledScaffold_2236	889	3357	-	HIST2H3D(3of7)
**864210**	GapFilledScaffold_3928	5303	20255	+	MYLIPA
**564175**	GapFilledScaffold_2794	18503	35432	+	BX957278.1(1of2)
**363169**	GapFilledScaffold_53	125.988	155.217	+	SI:DKEY-11F4.16(2of3)
**991287**	GapFilledScaffold_3820	32840	33602	+	NFURLNR03820030010
**919000**	GapFilledScaffold_51	100130	120451	+	NADL1.2(3of5)
**481517**	GapFilledScaffold_2326	84618	92286	+	TJAP1(3of3)
**58493**	GapFilledScaffold_8	285287	286965	-	CHAC1(1of2)
**316473**	GapFilledScaffold_2195	45195	47771	-	CDKN1A(2of2)
**751867**	GapFilledScaffold_2748	24178	38428	-	SI:DKEYP-67G8.1(1of2)
**982832**	GapFilledScaffold_2195	49180	49393	-	NFURLNR02195030100
**305262**	GapFilledScaffold_10553	5468	19187	-	KANSL3(1of7)
**18846**	GapFilledScaffold_10703	10568	15191	+	RYR3(4of9)
**662548**	GapFilledScaffold_2341	53173	63032	-	SI:CH211-119O8.4
**441154**	GapFilledScaffold_12511	2	9819	-	KANSL3(5of7)
**596594**	GapFilledScaffold_599	47298	49893	-	KLF11B
**153949**	GapFilledScaffold_2546	3968	18002	-	SI:CH73-209E20.3
**220820**	GapFilledScaffold_175	150807	164720	+	GLS2(2of2)
**84852**	GapFilledScaffold_1550	56556	63420	+	SI:CH211-212K5.3(4of8)
**497982**	GapFilledScaffold_4914	10487	16841	+	ZFAND5A
**993139**	GapFilledScaffold_51	81133	81439	+	NFURLNR00051020010
**587497**	GapFilledScaffold_14653	1836	5775	-	NFURG14653010002
**764839**	GapFilledScaffold_106	131576	143958	+	MKNK2B
**81394**	GapFilledScaffold_11811	6201	10887	-	POLR3GL
**147114**	GapFilledScaffold_6676	13656	16427	+	RTL1(5of18)
**636187**	GapFilledScaffold_9404	8566	20566	+	CADM4(1of3)
**307238**	GapFilledScaffold_5847	47948	75485	-	TSC22D3(3of3)
**338123**	GapFilledScaffold_1484	31465	43511	+	MNT(1of2)
**117280**	GapFilledScaffold_399	172004	185900	-	MNT(2of2)
**615046**	GapFilledScaffold_11090	1105	1416	-	HIST1H4I(1of10)
**544560**	GapFilledScaffold_1712	217259	228495	-	NR1D2(4of4)
**661977**	GapFilledScaffold_11895	4967	9139	-	NFURG11895010002
**265564**	GapFilledScaffold_713	145272	176766	-	NR4A3
**513443**	GapFilledScaffold_1396	169	2800	-	AP001468.1(14of32)
**779044**	GapFilledScaffold_4454	124114	124962	+	SI:CH73-269M14.4(7of7)
**351918**	GapFilledScaffold_28	341424	347751	-	GPR157
**483323**	GapFilledScaffold_3743	4748	7446	-	PLAUR(2of2)
**908266**	GapFilledScaffold_283	130842	138394	-	PELO
**973345**	GapFilledScaffold_574	307091	312611	+	BCL2L1
**631579**	GapFilledScaffold_3493	47800	53477	-	FAM107B
